# Probabilistic Estimation and Control of Dynamical Systems Using Particle Filter with Adaptive Backward Sampling

**DOI:** 10.3390/e26080653

**Published:** 2024-07-30

**Authors:** Taketo Omi, Toshiaki Omori

**Affiliations:** 1 Department of Electrical and Electronic Engineering, Graduate School of Engineering, Kobe University, 1-1 Rokkodai-cho, Nada-ku, Kobe 657-8501, Japan; take.me.1010.business@gmail.com; 2 Center for Mathematical and Data Sciences, Kobe University, 1-1 Rokkodai-cho, Nada-ku, Kobe 657-8501, Japan

**Keywords:** statistical machine learning, data assimilation, data-driven science, nonlinear dynamics, modern control theory

## Abstract

Estimating and controlling dynamical systems from observable time-series data are essential for understanding and manipulating nonlinear dynamics. This paper proposes a probabilistic framework for simultaneously estimating and controlling nonlinear dynamics under noisy observation conditions. Our proposed method utilizes the particle filter not only as a state estimator and a prior estimator for the dynamics but also as a controller. This approach allows us to handle the nonlinearity of the dynamics and uncertainty of the latent state. We apply two distinct dynamics to verify the effectiveness of our proposed framework: a chaotic system defined by the Lorenz equation and a nonlinear neuronal system defined by the Morris–Lecar neuron model. The results indicate that our proposed framework can simultaneously estimate and control complex nonlinear dynamical systems.

## 1. Introduction

Estimating dynamical systems from noisy time-series data is vital to understanding complex systems [1]. Furthermore, controlling dynamical systems has attracted significant attention in various fields for manipulating known complex systems. Controlling unknown complex systems requires simultaneously estimating and controlling dynamical systems from time-series data.

The state-space model has been utilized in various fields to estimate latent variables from noisy observational data [2,3,4,5,6,7,8,9,10]. The state-space model (SSM), also called the general hidden Markov model, is a probabilistic framework used to represent the dynamical system governing the latent variable and the probabilistic observation producing the observation variable. Under the assumption that the parameters governing the entire dynamics are known, the latent variable can be estimated efficiently using a sequential Bayesian filter such as a particle filter [11,12,13]. Nonetheless, in practice, it is unrealistic that the parameter values are completely known. If the parameters are unknown, it is necessary to estimate the latent variable and the parameters simultaneously. Therefore, many parameter estimation methods based on the particle filter have been proposed to estimate the latent variable and the parameters of the SSM (see [14] and the references therein). Some offline methods have been proposed, where every recursive parameter update requires a batch of observational data [15]. Other online methods have been proposed, where the parameter values can be estimated at the same time that new observational data become available [16]. However, previous online parameter estimation methods require multiple samples for each particle drawn from backward ancestor sampling every time, which leads to high computational costs.

In contrast, model predictive control has been applied in various fields to control nonlinear dynamical systems [17]. Model predictive control is a control strategy that predicts a known model’s behavior in a future interval and calculates an optimal control input; however, to apply model predictive control to a nonlinear model, it is necessary to formulate an optimal control problem, depicted by the calculus of variations or the Euler–Lagrange equation. Solving the optimal control problem, including nonlinearity, usually requires high computational cost and is not always feasible. In particular, particle filter-based model predictive control (PF-MPC) has been proposed [18]. PF-MPC can directly handle known nonlinear models by using a particle filter; however, in previous studies on control, the model dynamics are assumed to be known [18]. For considering real-world applications, the dynamics of complex systems are often unknown.

In this paper, we propose a framework for simultaneously estimating the latent variable and the parameters of the state-space model, as well as controlling the latent variable, fully based on the particle filter. The proposed method realizes a general method for estimating and controlling dynamical systems described by the state-space model by integrating and adapting the framework of the particle filter (PF), the online expectation-maximization (EM) algorithm, and model predictive control (MPC). With the PF, we estimate the latent variable of the nonlinear dynamics online. In particular, to fully realize the online algorithm for simultaneous estimation and control, we derive a novel online parameter estimation method for the state-space model by applying an adaptive smoothing (AdaSmooth) algorithm, which has recently been proposed for smoothing statistics based on the PF [19]. By combining AdaSmooth with the online EM algorithm, we efficiently approximate the sufficient statistics and estimate the parameter values based on the smoothed sufficient statistics. Furthermore, we consider combining PF-based model predictive control (PF-MPC) [18] with our AdaSmooth-based online EM algorithm to form the feedback control law. By introducing the PF not only as a state estimator and a prior estimator for the dynamics but also as a controller, we can directly handle the nonlinearity of the dynamics and uncertainty of the latent state.

This paper is organized as follows. Section 2 introduces the state-space model and the particle filter. We then propose the AdaSmooth-based online EM algorithm for estimating the parameters of the state-space model. Finally, by combining PF-MPC with the state estimation PF and the AdaSmooth-based online EM algorithm, we propose a framework for estimating and controlling dynamical systems. In Section 3, we verify the effectiveness of our proposed method using simulation environments. We apply our proposed framework to two distinct dynamical systems: a chaotic system defined by the Lorenz equation and a nonlinear neuronal model. In each subsection, we first formulate the state-space model for each dynamical system. We then derive the parameter estimation procedure based on the AdaSmooth-based online EM algorithm. Next, we derive the concrete control law based on PF-MPC before applying our proposed framework to each complex nonlinear dynamical system. Futhermore, we present the results for estimating and control the chaostic system and neural system. Finally, Section 4 presents our concluding remarks.

## 2. Methods

**Figure 1:**
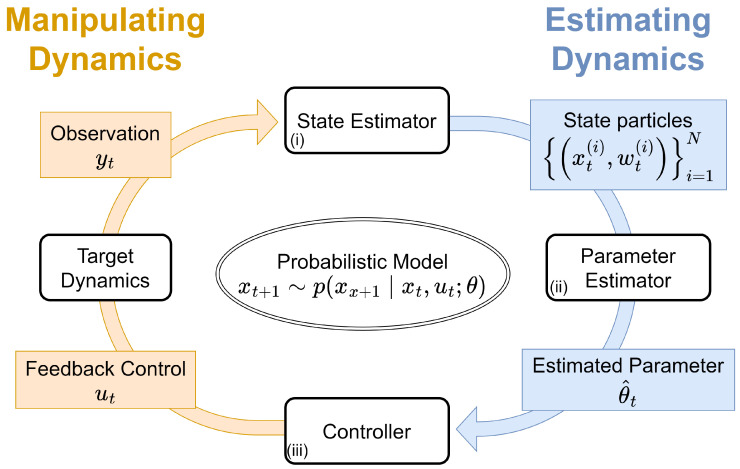
Overview of the proposed framework for probabilistic estimation and control of dynamical systems. (i) We apply a particle filter to estimate the latent state $x_{t}$ from noisy observational data $y_{t}$ online using data assimilation. (ii) We apply our adaptive smoothing (AdaSmooth)-based online expectation-maximization (EM) algorithm to estimate the dynamics parameter θ using the particles obtained from the state estimator in statistical machine learning. (iii) We employ another particle filter based on model predictive control to determine the approximate feedback control signal $u_{t}$ for controlling the target dynamics.

This study proposes a probabilistic framework for concurrent data assimilation-based control of dynamical systems. To handle a realistic situation, we assume that we can only observe noisy data via some observation model, i.e., we cannot directly obtain the true latent state. Figure 1 shows the overview of our proposed framework. Our proposed framework comprises three parts: (i) estimation of the posterior distribution of the latent state from noisy observational time-series data, (ii) online estimation of the model parameters governing the dynamical system, and (iii) solving the optimal control problem using a control PF with the estimated state and parameters.

### 2.1. Formulation of State-Space Model

**Figure 2:**
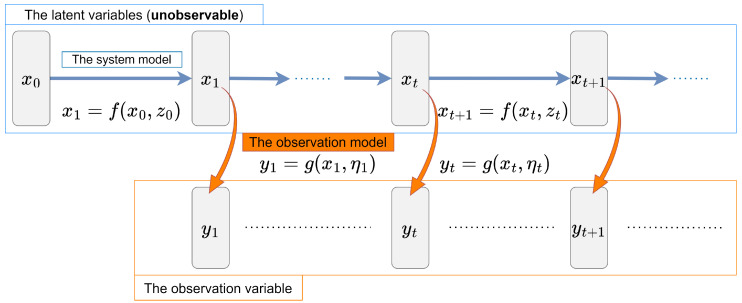
The overview of the state-space model (SSM).

First, we formulate the SSM. Figure 2 shows the overview of the SSM. The SSM includes the system model and the observation model. The system model represents the dynamics of the latent variable $x_{t}$. Conversely, the observation model represents how we obtain the observation variable $y_{t}$ under a realistic situation where some noise exists.

We consider the system model, including state $x_{t}$, control input $u_{t}$, and certain system noise $z_{t}$, with a Markov property:

(1) $$x_{t}=f\left(x_{t-1},u_{t-1},z_{t-1};\theta \right),$$

where *t* denotes a discrete time index, θ is an unknown parameter governing the entire dynamics, and *f* is a function determining the dynamics, which can include nonlinearity. Corresponding to Equation (1), we also define the probabilistic representation of the system model for the SSM as follows:

(2) $$x_{t}∼p\left(x_{t}|x_{t-1},u_{t-1};\theta \right).$$

We then formulate the observation model for the SSM. To reflect a realistic scenario, we assume that we can only obtain the observation variable $y_{t}$ and not the latent variable $x_{t}$ directly at time *t*. Hence, we formulate the following observation model for the SSM:

(3) $$y_{t}=g\left(x_{t},\eta_{t};\theta \right),$$

where *g* is a function determining the information we obtain via the observation and $\eta_{t}$ represents certain noise. Corresponding to Equation (3), we also define the probabilistic representation of the observation model for the SSM:

(4) $$y_{t}∼p\left(y_{t}|x_{t};\theta \right).$$

For simplicity, we assume that the joint probability $p(x_{1:t},y_{1:t};\theta )$ belongs to the exponential family, which is a common assumption. Here, $x_{1:t}$ and $y_{1:t}$ represent the time-series of the latent variable and observation variable $x_{1:t}=\left\{x_{1},\ldots ,x_{t}\right\}$ and $y_{1:t}=\left\{y_{1},\ldots ,y_{t}\right\}$, respectively. For example, when both the latent state and observation variable are one-dimensional real numbers and the model is linear Gaussian, the state-space model is described as follows:

(5) $$\left\{\begin{array}{cc} x_{t} & =ax_{t-1}+bz_{t-1}\\ y_{t} & =cx_{t}+d\eta_{t} \end{array}\right.,$$

where $x_{t}$ and $y_{t}\in R$ are the latent state and observation variable, respectively; a,b,c, and d∈R are parameters; and $z_{t-1}$ and $\eta_{t}$ are noise terms, both of which obey standard Gaussian distributions N(0,1). In this linear Gaussian case, the probabilistic representation of the SSM is expressed as follows:

(6) $$\begin{array}{c} x_{t}∼N\left(x_{t}|ax_{t-1},b^{2}\right), \end{array}$$

(7) $$\begin{array}{c} y_{t}∼N\left(y_{t}|cx_{t},d^{2}\right), \end{array}$$

where $N(x|\mu ,\sigma^{2})$ denotes the Gaussian distribution with mean μ and variance $\sigma^{2}$.

### 2.2. Estimation of the Hidden State

To estimate the latent state $x_{t}$ from noisy time-series observational data $y_{1:t}=\left\{y_{1},y_{2},\ldots ,y_{t}\right\}$ online, we apply the particle filter (PF) [11,12,13,20].

Here, we estimate the latent variable using the filtering distribution $p(x_{t}|y_{1:t})$. In the PF, the filtering distribution is approximated online using particles $\left\{x_{t}^{\left(1\right)},\ldots ,x_{t}^{\left(N\right)}\right\}$ and their associated weights $\left\{w_{t}^{\left(1\right)},\ldots ,w_{t}^{\left(N\right)}\right\}$, where *N* is the number of particles. We calculate these particles by alternately performing two steps: a resampling step and a prediction step.

In the resampling step, we resample particles obtained previously based on their weights. Although many resampling schemes have been proposed, we use multinomial resampling [21,22]. Using a multinomial distribution $M\left(w_{t-1}^{\left(1\right)},\ldots ,w_{t-1}^{\left(N\right)}\right)$ with weights, the ancestor index $A_{t}^{\left(i\right)}$ for the *i*-th particle at time *t* is sampled as follows:

(8) $$A_{t}^{\left(i\right)}∼M\left(w_{t-1}^{\left(1\right)},\ldots ,w_{t-1}^{\left(N\right)}\right),$$

where $i\in \left\{1,2,\ldots ,N\right\}$ is the index of the particle and $M(w^{\left(1\right)},\ldots ,w^{\left(N\right)})$ denotes the multinomial distribution supported on {1,2,…,N}. Using the ancestor index $A_{t}^{\left(i\right)}$, the *i*-th particle ${\bar{x}}_{t}^{\left(i\right)}$ at time *t* is resampled as follows:

(9) $${\bar{x}}_{t}^{\left(i\right)}=x_{t}^{\left(A_{t}^{\left(i\right)}\right)}.$$

We apply this resampling step only when the estimated effective sample size ${\hat{N}}_{\mathrm{eff}}$ satisfies ${\hat{N}}_{\mathrm{eff}}\left(t\right)\leq \alpha N$, where 0≤α≤1 is a hyperparameter that determines the threshold of the effective sample size [23]. Here, the estimated effective sample size ${\hat{N}}_{\mathrm{eff}}$ is calculated as follows:

(10) $${\hat{N}}_{\mathrm{eff}}\left(t\right)=\frac{1}{\sum_{i=1}^{N}{\left(w_{t}^{\left(i\right)}\right)}^{2}}.$$

When ${\hat{N}}_{\mathrm{eff}}\left(t\right)>\alpha N$, instead of probabilistic resampling, as described in Equation (8), we simply set $A_{t}^{\left(i\right)}=i$ for all particles.

In the prediction step, we sample particles that approximate the filtering distribution when new observational data $y_{t}$ are obtained. First, we sample each particle using the system model:

(11) $$x_{t}^{\left(i\right)}∼p\left(x_{t}|x_{t-1}^{\left(A_{t}^{\left(i\right)}\right)}\right).$$

We then calculate the weight of each particle using the observation model.

(12) $${\tilde{w}}_{t}^{\left(i\right)}=w_{t-1}^{\left(A_{t}^{\left(i\right)}\right)}p\left(y_{t}|x_{t}^{\left(A_{t}^{\left(i\right)}\right)}\right),$$

where ${\tilde{w}}_{t}^{\left(i\right)}$ denotes an un-normalized weight, and we assume that the weights from the previous time step and the ancestors are accessible. Note that the weight update given by Equation (12) is derived from sequential importance sampling [20], where the importance distribution is assumed to be the same as in the system model. Finally, we normalize the weights as follows:

(13) $$w_{t}^{\left(i\right)}=\frac{{\tilde{w}}_{t}^{\left(i\right)}}{\sum_{l=1}^{N}{\tilde{w}}_{t}^{\left(l\right)}}.$$

Using the sampled particles and their weights ${\left\{\left(x_{t}^{\left(i\right)},w_{t}^{\left(i\right)}\right)\right\}}_{i=1}^{N}$ allows us to obtain the filtering distribution of the latent variable online as follows:

(14) $$p\left(x_{t}|y_{1:t}\right)=\sum_{i=1}^{N}w_{t}^{\left(i\right)}\delta \left(x_{t}-x_{t}^{\left(i\right)}\right),$$

where δ(·) is Dirac’s delta function. Here, the particles and their weights at time t=0 are generated as follows:

(15) $$x_{0}^{\left(i\right)}∼p_{0}\left(x\right),w_{0}^{\left(i\right)}=\frac{1}{N},$$

where $p_{0}\left(x\right)$ is the initial distribution.

We estimate the latent state online by alternately applying the resampling and prediction steps as new observational data arrive. Although the PF can suffer from the path-degeneracy problem, it can be mitigated using the resampling step. In this study, we apply multinomial resampling; however, other resampling methods can be used in our proposed framework. The particles and their associated weights ${\left\{\left(x_{t}^{\left(i\right)},w_{t}^{\left(i\right)}\right)\right\}}_{i=1}^{N}$ are used to estimate the latent state and the parameters, including a prior for the state in the controller, within our proposed framework.

### 2.3. Estimation of the Parameter

We utilize the particle filter-based online parameter estimation strategy to estimate the parameters governing the dynamics online. This subsection briefly introduces the original expectation-maximization (EM) algorithm [15] and particle-based online EM algorithm [14,16]. We then derive an efficient parameter estimation procedure using additive smoothing (AdaSmooth) [19].

#### 2.3.1. EM Algorithm

The EM algorithm [15] is a widely used offline parameter estimation method. In the EM algorithm, when we have a batch of observational data $\left\{y_{1},\ldots ,y_{T}\right\}$, we estimate the parameter sequence $\left\{{\hat{\theta }}_{1},\ldots ,{\hat{\theta }}_{k},\ldots \right\}$ by alternately conducting an E-step and an M-step, where *T* is the terminal time and *k* is the number of iterations.

In the E-step, we calculate the expectation of the joint probability $p(x_{1:T},y_{1:T};\theta )$ given the previous iteration parameter ${\hat{\theta }}_{k-1}$.

(16) $$\Phi_{1:T;{\hat{\theta }}_{k-1}}\left[L_{1:T}\left(\theta \right)\right]=E{\left[\log p\left(x_{1:T},y_{1:T};\theta \right)\right]}_{p(x_{1:T}|y_{1:T},{\hat{\theta }}_{k-1})},$$

where $E{\left[\cdot \right]}_{p(x_{1:T}|y_{1:T},{\hat{\theta }}_{k-1})}$ denotes the expectation of the joint filtering distribution with respect to $x_{1:T}$ given the observational data $y_{1:T}$ and the parameter ${\hat{\theta }}_{k-1}$. $L_{1:T}\left(\theta \right)$ corresponds to the log-likelihood of the joint probability $p(x_{1:T},y_{1:T};\theta )$. In the M-step, we update the estimation parameter at this iteration ${\hat{\theta }}_{k}$ as follows:

(17) $$\begin{array}{c} {\hat{\theta }}_{k}=\arg\max_{\theta }\Phi_{1:T;{\hat{\theta }}_{k-1}}\left[L_{1:T}\left(\theta \right)\right], \end{array}$$

where $\arg\max_{\theta }$ denotes the maximizer with respect to the parameter θ.

The EM algorithm is an appealing parameter estimation method; however, we need a batch of observational data, and the computational cost of repeating two steps as the size of the observational data increases cannot be ignored. Furthermore, it is difficult to calculate the expectation in the E-step in typical cases. Moreover, an online parameter estimation algorithm must be utilized to realize a simultaneous method for estimating and controlling nonlinear dynamics.

#### 2.3.2. AdaSmooth-Based Online EM Algorithm

In this subsection, we propose the AdaSmooth-based online EM algorithm for estimating the parameters of the SSM. To overcome the problems in the original EM algorithm, some online adaptations have been proposed [14,24,25]. In particular, a particle-based online EM algorithm for time-series state-space models has been proposed [16]. However, a previous parameter estimation method based on PaRIS [26] requires multiple samples for each particle drawn from backward ancestor sampling every time, which leads to high computational costs.

**Figure 3:**
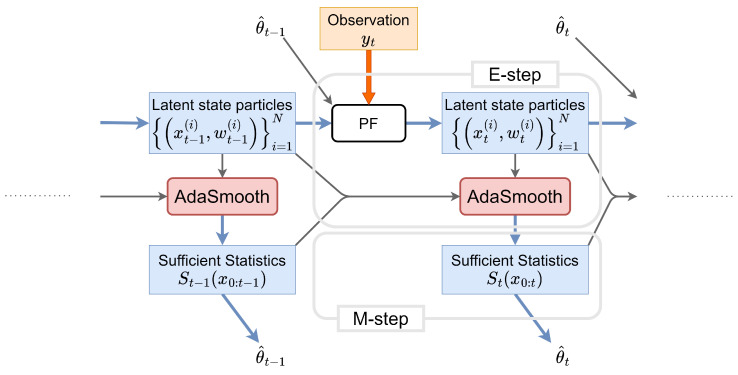
The flow of our proposed AdaSmooth-based online EM algorithm. (E-step) When the latent state particles ${\left\{\left(x_{t}^{\left(i\right)},w_{t}^{\left(i\right)}\right)\right\}}_{i=1}^{N}$ at time *t* are obtained by the particle filter (PF) with new observation $y_{t}$ and current parameter value ${\hat{\theta }}_{t-1}$, we update the sufficient statistics $S_{t}\left(x_{0:t}\right)$ using AdaSmooth, which incorporates particles at two subsequent times: t−1 and *y*${\left\{\left(x_{t-1}^{\left(i\right)},w_{t-1}^{\left(i\right)}\right)\right\}}_{i=1}^{N},{\left\{\left(x_{t}^{\left(i\right)},w_{t}^{\left(i\right)}\right)\right\}}_{i=1}^{N}$. (M-step) Based on the updated sufficient statistics $S_{t}\left(x_{0:t}\right)$, we estimate the parameter value ${\hat{\theta }}_{t}$ online. We can efficiently estimate dynamics online by repeating the E-step and the M-step as new observational data are obtained.

Figure 3 shows the flow of our proposed online EM algorithm for estimating the parameter $\theta_{t}$ online. We consider the parameter $\theta_{t}$ at time *t* rather than the iteration number *k* used in the offline EM algorithm. Note that the model parameter θ in the true dynamics is not time-varying, whereas the model parameter for estimating and controlling the dynamical system should be treated as a time-varying state parameter ${\hat{\theta }}_{t}$ in an online manner. Furthermore, we approximate smoothing sufficient statistics $\Phi_{1:t;{\hat{\theta }}_{t-1}}\left[S_{t}\right]$ using particles obtained online before and after time ${\left\{\left(x_{t-1}^{\left(i\right)},w_{t-1}^{\left(i\right)}\right)\right\}}_{i=1}^{N},{\left\{\left(x_{t}^{\left(i\right)},w_{t}^{\left(i\right)}\right)\right\}}_{i=1}^{N}$ via the PF [16]. To efficiently estimate the parameters, we apply AdaSmooth [19] to smooth the sufficient statistics. AdaSmooth requires only one sample for each particle drawn from backward ancestor sampling. In addition, AdaSmooth decides whether we should conduct backward ancestor sampling adaptively; hence, we can obtain approximated smoothing sufficient statistics $\Phi_{1:t;{\hat{\theta }}_{t-1}}\left[S_{t}\right]$ and estimate the parameter ${\hat{\theta }}_{t}$ online.

First, we rewrite the M-step in the EM algorithm. Assuming that the joint probability $p(x_{1:t},y_{1:t};\theta )$ belongs to the exponential family, a suitable function Λ(·) [14] exists as a maximizer of the log-likelihood in a specific form. That is, we can rewrite the M-step in the context of online estimation as follows:

(18) $${\hat{\theta }}_{t}=\Lambda \left(\frac{1}{t}\Phi_{1:t;{\hat{\theta }}_{t-1}}\left[S_{t}\right]\right),$$

where $\Phi_{1:t;{\hat{\theta }}_{t-1}}$ denotes the expectation with respect to the joint probability $p(x_{1:t}|y_{1:t},{\hat{\theta }}_{t-1})$. The parameter index now becomes time *t*, contrasting with the iteration count *k* used in Equation (17) in the offline context. $S_{t}$ is the sufficient statistics defined as follows:

(19) $$S_{t}\left(x_{0:t}\right)=\sum_{\tau =1}^{t}{\tilde{s}}_{\tau }\left(x_{\tau -1:\tau }\right).$$

Here, ${\tilde{s}}_{\tau }\left(x_{\tau -1:\tau }\right)={\tilde{s}}_{\tau }\left(x_{\tau -1},x_{\tau }\right)$ is a statistic given by the states $x_{\tau -1}$ and $x_{\tau }$ before and after times τ−1,τ.

Next, we estimate $\Phi_{1:t;{\hat{\theta }}_{t-1}}\left[S_{t}\right]$ online. At time *t*, we calculate the approximated smoothed sufficient statistics in the E-step [14,16] as follows:

(20) $$\Phi_{0:t;{\hat{\theta }}_{t-1}}\left[S_{t}\left(x_{0:t}\right)\right]=\int \kappa_{t}\left(x_{t}\right)p\left(x_{t}|y_{1:t}\right)dx_{t},$$

where $\kappa_{t}\left(x_{t}\right)$ is a statistic updated each time via the following procedure:

(21) $$\kappa_{t}\left(x_{t}\right)=\int \left\{\gamma_{t}{\tilde{s}}_{t}\left(x_{t-1},x_{t}\right)+\left(1-\gamma_{t}\right)\kappa_{t-1}\left(x_{t-1}\right)\right\}p\left(x_{t-1}|y_{1:t-1},{\hat{\theta }}_{1:t-1}\right)dx_{t-1},$$

where $\kappa_{0}\left(x_{0}\right)=0$ and $\gamma_{t}$ is a decay rate that satisfies $\sum_{t=1}^{\infty }\gamma_{t}=\infty$ and $\sum_{t=1}^{\infty }\gamma_{t}^{2}<\infty$.

Here, we estimate $\kappa_{t}\left(x_{t}\right)$ using particles. We prepare a new set of particles $\kappa_{t}\left(x_{t}\right)$. Then, we derive the update dynamics of $\kappa_{t}\left(x_{t}\right)$ based on AdaSmooth. Essentially, we update each particle $\kappa_{t}^{\left(i\right)}$ corresponding to Equation (21) as follows:

(22) $$\kappa_{t}^{\left(i\right)}=\left(1-\gamma_{t}\right)\kappa_{t-1}^{\left(i\right)}+\gamma_{t}{\tilde{s}}_{t}\left(x_{t-1}^{\left(A_{t}^{\left(i\right)}\right)},x_{t}^{\left(i\right)}\right),$$

where $A_{t}^{\left(i\right)}$ is the ancestor index obtained in the PF resampling step. The first term in Equation (22) represents the value at a previous time. The second term in Equation (22) represents the sufficient statistics, including the information obtained at time *t*. Nonetheless, updating $\kappa_{t}^{\left(i\right)}$ using only Equation (22) can lead to the particle path-degeneracy phenomenon and usually leads to instability. We introduce adaptive ancestor backward resampling to avoid this problem [19]. During resampling in the PF, when the diversity of the ancestors ${\hat{N}}_{\mathrm{anc}}\left(t\right)$ satisfies ${\hat{N}}_{\mathrm{anc}}\left(t\right)\leq \beta N$, we conduct backward ancestor resampling to sample $B_{t}^{\left(i\right)}$. We then update the dynamics using the following instead of Equation (22):

(23) $$\kappa_{t}^{\left(i\right)}=\frac{1}{2}\left\{\left(1-\gamma_{t}\right)\kappa_{t-1}^{\left(i\right)}+\gamma_{t}{\tilde{s}}_{t}\left(x_{t-1}^{\left(A_{t}^{\left(i\right)}\right)},x_{t}^{\left(i\right)}\right)\right\}+\frac{1}{2}\left\{\left(1-\gamma_{t}\right)\kappa_{t-1}^{\left(i\right)}+\gamma_{t}{\tilde{s}}_{t}\left(x_{t-1}^{\left(B_{t}^{\left(i\right)}\right)},x_{t}^{\left(i\right)}\right)\right\}.$$

The first term in Equation (23) is the same as in Equation (22). The second term in Equation (23) represents the correction of the sufficient statistics using a new ancestor drawn from backward ancestor sampling. Here, 0≤β≤1 is a hyperparameter that determines the threshold of ancestor diversity. ${\hat{N}}_{\mathrm{anc}}\left(t\right)$ is the number of unique Enoch indices $\left\{E_{t}^{\left(i\right)}\right\}$, defined as follows [19]:

(24) $${\hat{N}}_{\mathrm{anc}}\left(t\right)=\left|\{E_{t}^{\left(1\right)},\ldots ,E_{t}^{\left(N\right)}\}\right|.$$

Here, the Enoch index $E_{t}^{\left(i\right)}$ is updated via the following procedure:

(25) $$E_{t}^{\left(i\right)}=\left\{\begin{array}{cc} i & \left(t=t_{0}+1\right)\\ E_{t-1}^{\left(A_{t}^{\left(i\right)}\right)} & \left(t>t_{0}+1\right) \end{array}\right.,$$

where $t_{0}$ represents the last time backward ancestor sampling was conducted. The definition of each Enoch index shows that it retains the index of the ancestor at the last time backward ancestor sampling was conducted. Hence, a decrease in ${\hat{N}}_{\mathrm{anc}}\left(t\right)$ corresponds to a decrease in the diversity of the ancestors. The value of ${\hat{N}}_{\mathrm{anc}}\left(t\right)$ is used to determine whether we should conduct backward ancestor sampling.

Here, we show the procedure used in backward ancestor sampling to obtain the backward ancestor index $B_{t}^{\left(i\right)}$. According to Bayes’ theorem, the backward ancestor index is sampled as follows:

(26) $$B_{t}^{\left(i\right)}∼M\left({\left\{w_{t-1}^{\left(l\right)}p\left(x_{t}^{\left(i\right)}|x_{t-1}^{\left(l\right)}\right)\right\}}_{l=1}^{N}\right).$$

We implement this sampling scheme efficiently using rejection-acceptance sampling [19,26].

Once $\kappa_{t}\left(x_{t}\right)$ is approximated, the smoothing sufficient statistics are estimated as follows:

(27) $$\Phi_{0:t;{\hat{\theta }}_{t-1}}\left[S_{t}\left(x_{0:t}\right)\right]≃\sum_{i=1}^{N}w_{t}^{\left(i\right)}\kappa_{t}^{\left(i\right)}.$$

Finally, we estimate the parameter using Equation (18). The parameter ${\hat{\theta }}_{t}$, estimated using our AdaSmooth-based online EM algorithm, is also used in the controller described in Section 2.4.

Here, we briefly compare our proposed AdaSmooth-based online EM algorithm with the offline EM algorithm and their variants without considering control contexts. The offline EM algorithm requires many iterations with a batch of data. As a result, it has high computational costs. On the other hand, our AdaSmooth-based online EM algorithm requires no iteration for each step. Hence, our proposed framework realizes efficient online estimation of model parameters.

### 2.4. Control Strategy for the Dynamics

To design an efficient controller for the general state-space model, PF-based model predictive control (PF-MPC) [18] is integrated with our dynamics estimator in the proposed method. Model predictive control (MPC) is a theoretical framework used for feedback control in dynamical systems. In conventional MPC, an optimal control problem is formulated at time *t* over a future interval called a horizon, using the model $x_{t+1}=f\left(x_{t},u_{t}\right)$. Next, the problem is numerically solved to obtain the optimal control input series ${\left\{u_{\tau }^{*}\right\}}_{\tau \in \mathrm{horizon}}$ over the horizon. Finally, the first control input from this series is adopted as the actual input. Specifically, in conventional MPC, the optimal control problem in the horizon is treated as solving the Euler–Lagrange equation numerically, which is computationally expensive because it includes the derivative of the model concerning the state $x_{t}$ and control input $u_{t}$. In contrast, in PF-MPC, the optimal control problem is treated as a *filtering* problem for the control input $u_{t}$ based on an augmented state-space model within the horizon.

**Figure 4:**
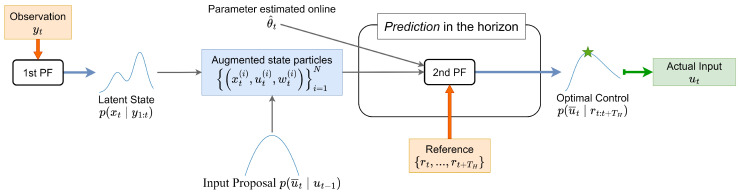
Overview of particle filter-based model predictive control (PF-MPC) integrated with our AdaSmooth-based online EM algorithm. The particle filter for state estimation (state PF) provides the distribution of latent variables $p(x_{t}|y_{1:t})$ as particles and their corresponding weights ${\left\{\left(x_{t}^{\left(i\right)},w_{t}^{\left(i\right)}\right)\right\}}_{i=1}^{N}$. These particles and their weights ${\left\{\left(x_{t}^{\left(i\right)},w_{t}^{\left(i\right)}\right)\right\}}_{i=1}^{N}$, obtained via the state PF, are directly used as initial augmented particles by the PF for control in the horizon (control PF). The input particles ${\left\{u_{t}^{\left(i\right)}\right\}}_{i=1}^{N}$ are sampled from the proposal distribution $p({\bar{u}}_{t}|u_{t-1})$. Using the control PF, we can obtain the filtering distribution of the optimal control $p({\bar{u}}_{t}|r_{t:t+T_{H}})$ given the reference trajectory; the optimal control input is calculated using a specific point estimator like the posterior mean.

Figure 4 shows the overview of PF-MPC. Our framework has two distinct PF modules: one (state PF) acts as a state estimator, as described in Section 2.2, and the other (control PF) serves as a solver for the control-filtering problem defined below. First, the latent state particles and their associated weights ${\left\{\left(x_{t}^{\left(i\right)},w_{t}^{\left(i\right)}\right)\right\}}_{i=1}^{N}$ are obtained via the PF. Then, the control input particles $\left\{{\bar{u}}_{t}^{\left(i\right)}\right\}$ are sampled from the proposal distribution $p({\bar{u}}_{t}|u_{t-1})$. Subsequently, by regarding the input variable as part of the latent state, we form augmented state particles ${\left\{\left(x_{t}^{\left(i\right)},{\bar{u}}_{t}^{\left(i\right)},w_{t}^{\left(i\right)}\right)\right\}}_{i=1}^{N}$ over the horizon. With the given reference trajectory $\left\{r_{t},\ldots ,r_{T+T_{H}}\right\}$, we solve the control-filtering problem by applying the control PF within the horizon and obtain the filtering distribution of the control input $p({\bar{u}}_{t}|r_{t},\ldots ,r_{T+T_{H}})$. Here, $T_{H}$ is the duration of the horizon. Finally, we adopt $u_{t}$ as the actual input based on the filtering distribution of the initial control input.

Here, we discuss the details of PF-MPC. To formulate the control-filtering problem, we define an augmented state-space model over the horizon. The augmented state-space model consists of the augmented system model and the augmented observation model.

First, we formulate the augmented system model over the horizon. Within this horizon, we consider the control input $u_{t}$ as part of the augmented state $\zeta_{t}=\left({\bar{x}}_{t},{\bar{u}}_{t},{\tilde{u}}_{t}\right)$, where ${\tilde{u}}_{t}$ serves as an auxiliary variable to preserve the initial input throughout the horizon. Here, ${\bar{x}}_{t},{\bar{u}}_{t}$, respectively, denote the predictive state and control input within the horizon. Note that the ${\bar{x}}_{t}$ and ${\bar{u}}_{t}$ notations are required to distinguish the predicted state and control input transitions over the horizon $\left\{\left({\bar{x}}_{t},{\bar{u}}_{t}\right),\ldots ,\left({\bar{x}}_{t+T_{H}},{\bar{u}}_{t+T_{H}}\right)\right\}$ from the actual state and input time series $\left\{\left(x_{1},u_{1}\right),\ldots ,\left(x_{t},u_{t}\right),\ldots \right\}$. Therefore, we define the following augmented system model for the augmented state $\zeta_{t}$ within the horizon:

(28) $$\zeta_{\tau +1}∼p\left(\zeta_{\tau +1}|\zeta_{\tau };{\hat{\theta }}_{t}\right)=p\left({\bar{x}}_{\tau +1}|{\bar{x}}_{\tau },{\bar{u}}_{\tau };{\hat{\theta }}_{t}\right)p\left({\bar{u}}_{\tau +1}|\zeta_{t}\right)\delta \left({\tilde{u}}_{\tau +1}-{\tilde{u}}_{\tau }\right),$$

where $\tau \in \{t,t+1,\ldots ,t+T_{H}\}$ is the time index within the horizon, $p({\bar{x}}_{\tau +1}|{\bar{x}}_{\tau },{\bar{u}}_{\tau };{\hat{\theta }}_{t})$ is defined using the original system model within the state-space model described in Section 2.1, $p({\bar{u}}_{\tau +1}|\zeta_{\tau })$ is the transition probability, and $\delta ({\tilde{u}}_{\tau +1}-{\tilde{u}}_{\tau })$ is the probability corresponding to the deterministic transition ${\tilde{u}}_{\tau +1}={\tilde{u}}_{\tau }$. The parameter ${\hat{\theta }}_{t}$ is estimated using our AdaSmooth-based online EM algorithm and is fixed throughout the horizon.

Next, we formulate the augmented observation model. We consider controlling the latent state $x_{t}$ toward the given reference trajectory $\left\{r_{1},\ldots ,r_{t},\ldots \right\}$. We define the augmented observation model as follows:

(29) $$r_{\tau }∼p\left(r_{\tau }|\zeta_{\tau }\right).$$

For example, a Gaussian distribution can be used as the observation model:

(30) $$r_{\tau }∼N\left(r_{\tau }|R_{\tau }\zeta_{\tau },\Sigma_{r}\right),$$

where $R_{\tau }$ is an appropriately sized matrix and $\Sigma_{r}$ is a diagonal covariance matrix that adjusts the error between the state and reference trajectory. Note that the reference value’s dimension does not have to be the same as that of the latent variable, i.e., $R_{\tau }$ can be a non-square matrix. Furthermore, the augmented observation model is very flexible, but its details are omitted here (see [18]).

We then solve the control-filtering problem given by the augmented state-space model [Equations (28) and (29)] and the given reference trajectory ${\left\{r_{\tau }\right\}}_{\tau =t}^{t+T_{H}}$. Using control PF in the horizon $\left\{t,t+1,\ldots ,t+T_{H}\right\}$, we solve the control-filtering problem efficiently to *estimate* the augmented state $\zeta_{t}=\left({\bar{x}}_{t},{\bar{u}}_{t},{\tilde{u}}_{t}\right)$. Here, the initial augmented particles ${\left\{\zeta_{t}^{\left(i\right)}=\left({\bar{x}}_{t}^{\left(i\right)},{\bar{u}}_{t}^{\left(i\right)},{\tilde{u}}_{t}^{\left(i\right)}\right)\right\}}_{i=1}^{N}$ and their associated weights ${\left\{{\bar{w}}_{t}^{\left(i\right)}\right\}}_{i=1}^{N}$ in the horizon are initialized for each particle using information obtained from the state PF as follows:

(31) $$\begin{array}{cc} {\bar{x}}_{t}^{\left(i\right)} & =x_{t}^{\left(i\right)}, \end{array}$$

(32) $$\begin{array}{cc} {\bar{u}}_{t}^{\left(i\right)} & ∼p({\bar{u}}_{t}^{\left(i\right)}|u_{t}^{\left(i\right)}), \end{array}$$

(33) $$\begin{array}{cc} {\tilde{u}}_{t}^{\left(i\right)} & ={\bar{u}}_{t}^{\left(i\right)}, \end{array}$$

(34) $$\begin{array}{cc} {\bar{w}}_{t}^{\left(i\right)} & =w_{t}^{\left(i\right)}, \end{array}$$

where ${\left\{\left(x_{t}^{\left(i\right)},w_{t}^{\left(i\right)}\right)\right\}}_{i=1}^{N}$ are the particles obtained from the state PF for the latent state estimation and $p({\bar{u}}_{t}^{\left(i\right)}|u_{t}^{\left(i\right)})$ is the initial proposal distribution for the control input. To avoid confusion, we note here that the aim of the control PF is to estimate the optimal control input obtained using the initial control input via the proposal distribution. For filtering the initial control input ${\bar{u}}_{t}$, an auxiliary variable ${\tilde{u}}_{t}$ is defined to preserve the initial input ${\bar{u}}_{t}$. Although ${\tilde{u}}_{t}^{\left(i\right)}={\bar{u}}_{t}^{\left(i\right)}$ at time *t* is defined in Equation (31), the predictive control inputs ${\bar{u}}_{t+1}^{\left(i\right)},{\bar{u}}_{t+2}^{\left(i\right)},\ldots$ in the horizon have values different from ${\tilde{u}}_{t}^{\left(i\right)}$. Therefore we need both notations ${\bar{u}}_{t}$ and ${\tilde{u}}_{\tau }$ for the control PF in the horizon.

Finally, we estimate the optimal control input based on the filtered particles. By extracting the auxiliary variables that retain the initial control input and their associated weights ${\left\{\left({\tilde{u}}_{t+T_{H}}^{\left(i\right)},{\bar{w}}_{t+T_{H}}^{\left(i\right)}\right)\right\}}_{i=1}^{N}$, we compute the actual control input at time *t* via point estimation of the filtering distribution as follows:

(35) $$u_{t}=\sum_{i=1}^{N}{\bar{w}}_{t+T_{H}}^{\left(i\right)}{\tilde{u}}_{t+T_{H}}^{\left(i\right)}.$$

It should be noted that the auxiliary variable ${\tilde{u}}_{t+T_{H}}$ inherits the information at time $t,\ldots ,t+T_{H}-1$. Hence, to obtain the estimated optimal control input, we focus solely on these particles at time $t,\ldots ,t+T_{H}-1$ within the horizon.

Here, we briefly discuss the differences between conventional MPC and PF-MPC. MPC requires derivatives of the model concerning the state and the control input. Furthermore, exact point estimation of the latent state is required. Conversely, PF-MPC does not require model derivatives and only relies on particles representing the latent variable. The only requirement of PF-MPC is that the model can be simulated forward in time, which is satisfied in typical cases. Nonetheless, under certain assumptions, the control input obtained using PF-MPC is equivalent to that of conventional MPC [18]. Moreover, to clarify the advantage of our proposed method compared with previous work, we briefly touch on the differences between the previous work [27] and our proposed framework. Three significant differences exist between the previous work and our proposed framework. First, the previous work updates parameters at fixed intervals. In contrast, our proposed framework updates parameters continuously, with the controller referencing these updated parameters; hence, our proposed framework is more adaptive. Second, the previous work uses the offline EM algorithm for parameter estimation. Due to the structure of the offline EM algorithm, the previous framework maintains a history of particles within a window. In contrast, our proposed method is based on the online EM algorithm. Using the online EM algorithm as the fundamental parameter estimation strategy, we retain only the preceding and succeeding particle sets; therefore, our framework is more memory-efficient. Third, in the previous work, there are complex hyperparameters, e.g., the parameter update interval and window length. These complex hyperparameters require sensitive hand-tuning and domain-specific knowledge. Conversely, the hyperparameters of our proposed framework are defined in a simpler form. For further details, the typical values of the hyperparameters for AdaSmooth are discussed in [19]. Therefore, our proposed framework is easier to tune compared with the previous work.

We have proposed a fully PF-based framework for simultaneously estimating the latent state and the parameters governing the dynamics, and controlling the dynamics against a general state-space model. Although conventional MPC can handle the model’s nonlinearity directly and PF-MPC can address the model’s uncertainty, the latent variable and dynamics are assumed to be known. The previous method [27] attempts to resolve this problem; however, it requires difficult hyperparameter tuning and applies only to neuronal models. Our proposed method establishes a feedback control law for general state-space models by combining a PF and an AdaSmooth-based online EM algorithm.

## 3. Experiments

We verify the effectiveness of our proposed method in a simulation environment. We apply our proposed method to two complex systems: the Lorenz system and the Morris–Lecar neuron model. For each distinct model, we first formulate its complex dynamics as a general SSM. Next, we derive the sufficient statistics necessary for our AdaSmooth-based online EM algorithm to estimate the parameters. Then, we formulate the augmented SSM over the horizon to control the dynamics. Finally, the experimental settings and results for each experiment are shown.

### 3.1. Application to Chaotic Lorenz System

The Lorenz system, introduced in [28], is known as a chaotic system. Because chaotic behavior is not always desirable, eliminating it is essential for practical application. Delayed feedback control has been proposed to stabilize chaotic models [29,30]. In contrast, a control strategy for chaotic behavior with simultaneous parameter estimation using statistical machine learning has not been studied. By applying our proposed method to the Lorenz system, we aim to confirm its ability to estimate the latent state and parameters of the chaotic Lorenz system and to stabilize its chaotic behavior.

The Lorenz system is given as follows:

(36) $$\left\{\begin{array}{cc} \frac{dx}{dt^{\prime }} & =\sigma (y-x),\\ \frac{dy}{dt^{\prime }} & =rx-y-xz+u,\\ \frac{dz}{dt^{\prime }} & =xy-bz, \end{array}\right.$$

where $t^{\prime }$ represents continuous time; ${\left(x,y,z\right)}^{⊤}\in R^{3}$ represents the three-dimensional latent states; *u* is the control input; and σ,r,b are parameters. Based on a previous study on suppressing chaotic behavior [30], we assume the control input only affects one element, *y*. To avoid confusion, the variables used in the Lorenz system are shown in Table 1. When we fix u=0, the Lorenz system with $\sigma =10,b=8/3,r>r_{H}$ exhibits subcritical Hopf bifurcation, where $r_{H}≃24.73684211$. We aim to control the *y*-element value toward the unstable fixed point $y_{f}=\sqrt{b(r-1)}$ under noisy environmental conditions of the latent state where only noisy observations of the latent state can be observed. To this end, we first derive the SSM for the Lorenz system. We then derive the E-step and the M-step of our online EM algorithm. Finally, we formulate the augmented SSM for the Lorenz system to control the latent state. With these Lorenz system formulations, we apply the proposed method to the Lorenz system.

#### 3.1.1. Formulation of State-Space Model

Here, we derive the SSM for the Lorenz system. We first derive the system model of the SSM. We discretize the continuous-time model given by Equation (36) with a time step Δt.

(37) $$x_{t+1}=x_{t}+f\left(x_{t},u_{t};\theta \right)\Delta t,$$

where $x_{t}={\left[x_{t},y_{t},z_{t}\right]}^{⊤}\in R^{3}$ corresponds to the latent state at time tΔt, $u_{t}\in R$ is the control input at time tΔt, and $\theta ={\left[\sigma ,r,b\right]}^{⊤}\in R^{3}$ corresponds to the unknown parameters of the system. $f(x_{t},u_{t};\theta )$ is a function that describes the dynamics of the Lorenz system as follows:

(38) $$f\left(x_{t},u_{t};\theta \right)=\left[\begin{array}{c} \sigma (y_{t}-x_{t})\\ rx_{t}-y_{t}-x_{t}z_{t}+u_{t}\\ x_{t}y_{t}-bz_{t} \end{array}\right].$$

Next, we introduce system noise governed by additive Gaussian noise with zero mean and covariance matrix $\Sigma_{x}=\mathrm{diag}\left(\sigma_{x_{1}}^{2},\sigma_{x_{2}}^{2},\sigma_{x_{3}}^{2}\right)\in R^{3\times 3}$. Here, $\mathrm{diag}(a_{1},a_{2},a_{3})$ denotes the diagonal matrix with diagonal elements $a_{1},a_{2},a_{3}$. The system model of the SSM for the Lorenz system is formulated as follows:

(39) $$p\left(x_{t+1}|x_{t},u_{t},\theta \right)=N\left(x_{t+1}|x_{t}+f\left(x_{t},u_{t};\theta \right)\Delta t,\Sigma_{x}\Delta t\right).$$

Next, we derive the observation model of the SSM. We assume that the latent state with additive Gaussian noise is obtained. Hence, we formulate the observation model as follows:

(40) $$y_{t}∼N\left(y_{t}|x_{t},\Sigma_{y}\right),$$

where $\Sigma_{y}\in R^{3\times 3}$ is the covariance matrix corresponding to the observation noise.

#### 3.1.2. Derivation of Online EM Algorithm

Here, we derive the sufficient statistics $S_{t}\left(x_{0:t}\right)$ and the update function Λ(·) for the Lorenz SSM, using Equations (39) and (40), in our AdaSmooth-based online EM algorithm. After calculation, we obtain the maximizer of the expectation of log-likelihood Λ(·) as follows:

(41) $$\Lambda \left(S_{t}=\left(A_{t},b_{t}\right)\right)=A_{t}^{-1}b_{t},$$

where the sufficient statistics $S_{t}=\left(A_{t},b_{t}\right)$ comprise two additive-form statistics $A_{t}\in R^{3\times 3}$ and $b_{t}\in R^{3}$, given as follows:

(42) $$\begin{array}{c} A_{t}\left(x_{0:t}\right)=\sum_{\tau =1}^{t}{\tilde{A}}_{\tau }\left(x_{\tau -1:\tau }\right), \end{array}$$

(43) $$\begin{array}{c} b_{t}\left(x_{0:t}\right)=\sum_{\tau =1}^{t}{\tilde{b}}_{\tau }\left(x_{\tau -1:\tau }\right), \end{array}$$

where ${\tilde{A}}_{\tau }$ and ${\tilde{b}}_{\tau }$ are defined as follows:

(44) $$\begin{array}{c} {\tilde{A}}_{\tau }\left(x_{\tau -1:\tau }\right)=\mathrm{diag}\left(\frac{{\left(y_{\tau -1}-x_{\tau -1}\right)}^{2}}{\sigma_{x_{1}}^{2}},\frac{x_{\tau -1}^{2}}{\sigma_{x_{2}}^{2}},\frac{z_{\tau -1}^{2}}{\sigma_{x_{3}}^{2}}\right)\Delta t, \end{array}$$

(45) $$\begin{array}{c} {\tilde{b}}_{\tau }\left(x_{\tau -1:\tau }\right)=\left[\begin{array}{c} \frac{y_{\tau -1}-x_{\tau -1}}{\sigma_{x_{1}}^{2}}\left(x_{\tau }-x_{\tau -1}\right)\\ \frac{x_{\tau -1}}{\sigma_{x_{2}}^{2}}\left\{y_{\tau }-y_{\tau -1}+\left(y_{\tau -1}+x_{\tau -1}z_{\tau -1}-u_{\tau -1}\right)\Delta t\right\}\\ -\frac{z_{\tau -1}}{\sigma_{x_{3}}^{2}}\left(z_{\tau }-z_{\tau -1}-x_{\tau -1}y_{\tau -1}\Delta t\right) \end{array}\right]. \end{array}$$

#### 3.1.3. Formulation of the Augmented State-Space Model for Control

We aim to suppress the chaotic behavior and control the *y*-element of the latent state toward the fixed point $y_{f}=\sqrt{b(r-1)}$ [30]. First, we define the control transition over the horizon. To consider realistic scenarios, we impose a constraint concerning the control input at every time *t* as follows:

(46) $$-u_{\lim}\leq u_{t}\leq u_{\lim},$$

where $u_{\lim}>0$ is the limit of the control input. Therefore, we define the augmented system model for the control input transition as follows:

(47) $${\bar{u}}_{\tau +1}={\mathrm{clamp}}_{u_{\lim}}\left(u_{\tau }+z_{\tau +1}\right),$$

where $z_{\tau +1}∼N\left(0,\sigma_{u}^{2}\right)$ is Gaussian noise and ${\mathrm{clamp}}_{u_{\lim}}\left(\cdot \right)$ is a clamp function defined as follows:

(48) $${\mathrm{clamp}}_{u_{\lim}}\left(u\right)=\frac{|u+u_{\lim}|-|u-u_{\lim}|}{2}.$$

To initialize the augmented particles, we define the following initialization procedure corresponding to the initial proposal distribution $p({\bar{u}}_{t}^{\left(i\right)}|u_{t}^{\left(i\right)})$:

(49) $${\bar{u}}_{t}^{\left(i\right)}={\mathrm{clamp}}_{u_{\lim}}\left(u_{t}+z_{t}^{\left(i\right)}\right),$$

where $z_{t}^{\left(i\right)}∼N\left(0,\sigma_{u0}^{2}\right)$ is Gaussian noise. Note that we do not need a probabilistic form of the augmented SSM.

We then define the augmented observation model of the augmented SSM over the horizon. We aim to control the y-element of the latent state toward the fixed point $y_{f}$; hence, we define the augmented observation model for the PF-MPC controller as follows:

(50) $$r_{\tau }∼N\left(r_{\tau }|y_{t},\sigma_{r}^{2}\right),$$

where $\sigma_{r}^{2}\in R$ is a variance that adjusts for acceptable error against the reference trajectory. The given reference value is also always equivalent to $y_{f}$, i.e., $r_{t}=y_{f}$ for t=1,2,….

#### 3.1.4. Settings

The true dynamics are assumed to be the Lorenz system defined by Equation (36), and in the simulation, they are solved using the Runge–Kutta method. Here, the true parameters are σ=10 and r=28,b=8/3. We apply our proposed method to the Lorenz system to suppress its chaotic behavior toward the unstable fixed point $y_{f}=\sqrt{b(r-1)}$. Note that we can only observe the noisy time series of the latent variable, not the true value.

The hyperparameters are set as follows. For simulation, the time step is Δt=0.01. The variances of the system noise and the observation noise are $\Sigma_{x}=\mathrm{diag}\left(\sigma_{x_{1}}^{2},\sigma_{x_{2}}^{2},\sigma_{x_{3}}^{2}\right)=\mathrm{diag}\left(1^{2},1^{2},1^{2}\right)$ and $\Sigma_{y}=\mathrm{diag}\left(1^{2},1^{2},1^{2}\right)$, respectively. For the PF, the number of particles is N=1000, and the threshold rate of resampling is α=0.8. The initial particles ${\left\{\left(x_{t}^{\left(i\right)},w_{t}^{\left(i\right)}\right)\right\}}_{i=1}^{N}$ are sampled from the initial distribution, defined as follows:

(51) $$p_{0}\left(x\right)=N\left(x|x_{0},\Sigma_{x0}\right),$$

where $\Sigma_{x0}=\mathrm{diag}\left(10^{2},10^{2},10^{2}\right)$. For parameter estimation, we use the threshold rate of backward resampling β=0.7 and the decay rate $\gamma_{t}=t^{-1}$. Following [16], we start updating the parameter after $T_{\mathrm{burnIn}}$ steps. In this experiment, we set $T_{\mathrm{burnIn}}=100$. To control the Lorenz system, we set the number of steps in the horizon to $T_{H}=10$. As a hyperparameter in the augmented SSM, the acceptable error variance and the control input limit are $\sigma_{r}^{2}=1^{2}$ and $u_{\lim}=10$, respectively. The variances of the augmented SSM’s control transitions are $\sigma_{u}^{2}=1^{2}$ and $\sigma_{u0}^{2}=10^{2}$.

#### 3.1.5. Results

**Figure 5:**
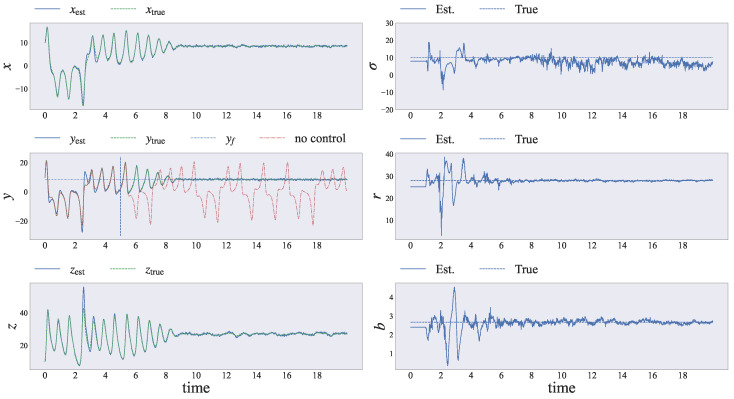
Results for simultaneous estimating and controlling the Lorenz system. The dynamical behavior of the state variables $x_{t}={\left[x_{t},y_{t},z_{t}\right]}^{⊤}$ and model parameters $\theta ={\left[\sigma ,r,b\right]}^{⊤}$ are shown. By applying the reference signal [fixed point $y_{f}=\sqrt{b(r-1)}$], all three variables (x,y,z) approach a steady point $\left(x_{f},y_{f},z_{f}\right)=\left(\sqrt{b(r-1)},\sqrt{b(r-1)},r-1\right)$. The latent state $\left[x_{t},y_{t},z_{t}\right]$ is estimated using our proposed method over time. In addition, all of the estimated parameters converge to the true values compared to the initial values, although σ oscillates slightly. To confirm that the model exhibits chaotic behavior, we provide the control input after time t=5 (gray dashed line in the middle-left graph).

Figure 5 shows the simulation results after applying our proposed framework to the Lorenz system. We confirmed that our method can simultaneously estimate and control the Lorenz dynamics. To verify whether our proposed method can suppress chaotic behavior, we did not adopt the control input until t=5.

The graphs on the left in Figure 5 show the x,y,z elements of the latent state from top to bottom, respectively. The state estimated by the PF represents the filtering distribution of the latent state. Hence, to confirm that we can estimate the latent variable, the estimated value was calculated using the weighted mean of the particles as follows:

(52) $$x_{\mathrm{est},t}=\sum_{i=1}^{N}w_{t}^{\left(i\right)}x_{t}^{\left(i\right)}.$$

The three graphs on the left in Figure 5 show the estimated values $\left(x_{\mathrm{est}},y_{\mathrm{est}},z_{\mathrm{est}}\right)$ (blue solid lines) and the true values $\left(x_{\mathrm{true}},y_{\mathrm{true}},z_{\mathrm{true}}\right)$ (green dashed lines), respectively. We found that the estimated values tracked the true values over time. In particular, the middle graph in Figure 5 also shows the target unstable fixed point $y_{f}$ (blue dotted line). We also found that the estimated values $\left(x_{\mathrm{est}},y_{\mathrm{est}},z_{\mathrm{est}}\right)$ accurately reproduced the true values $\left(x_{\mathrm{true}},y_{\mathrm{true}},z_{\mathrm{true}}\right)$, and the state was successfully manipulated. Here, the red dash-dotted line represents the simulation results when no input was applied, as shown in the graphs on the left in Figure 5. Without our proposed method, the Lorenz system still exhibited chaotic behavior. Conversely, after applying our proposed method, the Lorenz system stabilized and converged to the fixed point $y_{f}$.

**Figure 6:**
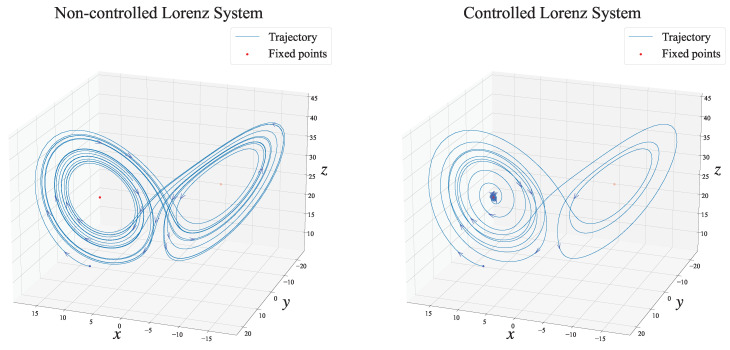
Dimensional visualization of trajectories with the non-controlled and controlled Lorenz systems. Left: The non-controlled trajectory obtained through simulation, starting with the same initial state used in the experiment. Right: The controlled trajectory obtained through simultaneous estimation and control of dynamics using our proposed method. Here, the arrows on each trajectory represent the direction of the transitions. By applying our proposed framework, the Lorenz system stabilizes around the desired fixed point $\left(x,y,z\right)=\left(\sqrt{b(r-1)},\sqrt{b(r-1)},r-1\right)$.

To confirm whether our proposed method could suppress chaotic behavior, we visualized the trajectory of the latent variable $\left(x_{t},y_{t},z_{t}\right)$ for the non-controlled and controlled Lorenz systems in a three-dimensional graph. Figure 6 shows the two trajectories of the non-controlled Lorenz system and the Lorenz system controlled by our proposed automatic control law. The left side of Figure 6 shows the simulation results of the true dynamics when the control input was fixed at zero: $u_{t}=0$. When we did not adopt any control input, the Lorenz system exhibited chaotic behavior around two fixed points. Conversely, by applying our proposed framework, as shown on the right in Figure 6, the Lorenz system stabilized around the fixed point determined in advance.

Next, we clarified whether our proposed method could estimate the unknown parameters. The graphs on the right in Figure 5 show the parameters governing the Lorenz system. Each estimated parameter value of three types $\left(\sigma_{t},r_{t},b_{t}\right)$ of the Lorenz system converged to the true values over time, although the estimated value for $\sigma_{t}$ oscillated slightly. In particular, the bifurcation parameter $r_{t}$, which influences the dynamics of *y*, was accurately estimated by our AdaSmooth-based online EM algorithm. Additionally, $b_{t}$, governing the dynamics of *z*, was also estimated accurately by our proposed method.

**Figure 7:**
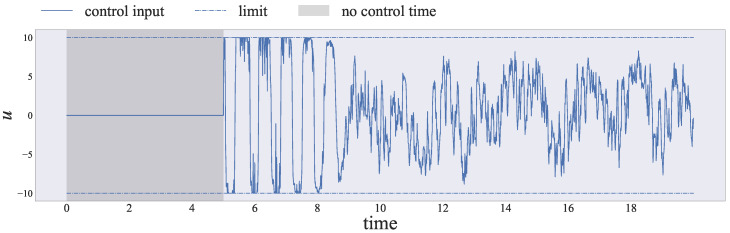
The history of the control input $u_{t}$ obtained automatically by our proposed method. Note that the control input $u_{t}$ always satisfies the absolute value constraint $|u_{t}|\leq u_{\lim}$. Nonetheless, Figure 5 shows that we can simultaneously estimate and control chaotic nonlinear dynamical systems.

Finally, we clarified whether our proposed method could estimate and control the Lorenz dynamics using the control input $u_{t}$ with a limited range of strengths. Figure 7 shows the control input (blue line) and the limit value (blue dash-dotted line). We maintained $u_{t}=0$ during the time period marked with a gray background. The control input $u_{t}$ always satisfied the norm constraint $|u_{t}|\leq u_{\lim}$. Hence, we can control the Lorenz dynamics simultaneously, even if we impose input constraints.

### 3.2. Application to Morris–Lecar Neuron Model

It is important to estimate and control the nonlinear dynamics of neurons to understand nerve systems and brain functions [31,32]. Nonetheless, it is difficult to control neuronal dynamics due to their complexity and partial observability. Typically, only noisy membrane potentials are observable [33,34,35].

Following previous work [27], this section aims to control the time series of membrane potentials toward a given reference trajectory under realistic conditions, where only noisy time series of membrane potentials can be observed. To this end, we first derive the SSM for the Morris–Lecar neuron model. We then derive the sufficient statistics and the update function for our online AdaSmooth-based EM algorithm for online parameter estimation. Finally, we formulate the augmented SSM for the Morris–Lecar neuron model to control the membrane potential. We apply the proposed method to the Morris–Lecar neuron model based on these SSM formulations.

#### 3.2.1. Formulation of State-Space Model

In the Morris–Lecar neuron model [36], the nonlinear dynamics of the membrane potential *v* and the channel variable *n* are described by the following continuous- time model:

(53) $$\left\{\begin{array}{cc} C_{m}\frac{dv}{dt^{\prime }} & =-g_{L}\left(v-E_{L}\right)-g_{Ca}m_{\infty }\left(v\right)\left(v-E_{Ca}\right)-g_{K}n\left(v-E_{K}\right)+I\\ \tau \left(v\right)\frac{dn}{dt^{\prime }} & =-n+n_{\infty }\left(v\right), \end{array}\right.$$

where *v* is the membrane potential, *n* is the channel variable, and *I* is the external input current. Here, $\tau \left(v\right)=\frac{1}{\phi }\cosh^{-1}\left(\frac{v-V_{3}}{2V_{4}}\right)$ is a function that determines the speed of the dynamics. $m_{\infty }\left(v\right)$ and $n_{\infty }\left(v\right)$ are nonlinear functions that describe the calcium and potassium dynamics; $E_{L},E_{Ca},$ and $E_{K}$ are the reversal potentials; and $C_{m}$ is the membrane capacitance [27]. To avoid confusion, the variables used in the Morris–Lecar neuron model are shown in Table 2.

First, we derive the system model for the SSM. The continuous-time model given by Equation (53) is discretized with a time step Δt.

(54) $$x_{t+1}=x_{t}+f\left(x_{t},u_{t};\theta \right)\Delta t,$$

where $x_{t}={\left[v_{t},n_{t}\right]}^{⊤}\in R^{2}$ corresponds to the latent state at time tΔt, $u_{t}\in R$ corresponds to the control input at time tΔt, and $\theta ={\left[g_{L},g_{Ca},g_{K}\right]}^{⊤}\in R^{3}$ corresponds to the unknown parameters. Following previous work [27], we separate the net input $I_{t}$ into the controllable input $u_{t}$ and the known streaming currents from other neurons $I_{t}^{\mathrm{inj}}$, i.e., $I_{t}=u_{t}+I_{t}^{\mathrm{inj}}$. $f(x_{t},u_{t};\theta )$ is a function representing the dynamics of the Morris–Lecar neuron model as follows:

(55) $$f\left(x_{t},u_{t};\theta \right)=\left[\begin{array}{c} -\frac{1}{C_{m}}\left\{g_{L}\left(v_{t}-E_{L}\right)+g_{Ca}m_{\infty }\left(v_{t}\right)\left(v_{t}-E_{Ca}\right)+g_{K}n\left(v_{t}-E_{K}\right)\right\}+\frac{1}{C_{m}}I_{t}\\ -\phi \cosh\left(\frac{v_{t}-V_{3}}{2V_{4}}\right)\left(n_{t}-n_{\infty }\left(v_{t}\right)\right) \end{array}\right].$$

We then introduce system noise governed by white Gaussian noise with zero mean and covariance matrix $\Sigma_{x}=\mathrm{diag}\left(\sigma_{v}^{2},\sigma_{n}^{2}\right)\in R^{2\times 2}$. The system model of the SSM for the neuron model is formulated as follows:

(56) $$p\left(x_{t+1}|x_{t},u_{t},\theta \right)=N\left(x_{t+1}|x_{t}+f\left(x_{t},u_{t};\theta \right)\Delta t,\Sigma_{x}\Delta t\right).$$

Next, we formulate the observation model for the SSM. We consider that only noisy membrane potentials $y_{t}\in R$ can be observed. Introducing additive Gaussian noise as observation noise, we formulate the observation model as follows:

(57) $$y_{t}∼N\left(y_{t}|v_{t},\sigma_{y}^{2}\right),$$

where $\sigma_{y}^{2}\in R$ is the variance corresponding to the observation noise.

#### 3.2.2. Derivation of Online EM Algorithm

Here, we derive the sufficient statistics $S_{t}\left(x_{0:t}\right)$ and the update function Λ(·) for the neuron model described by Equations (56) and (57), which are used in our AdaSmooth-based online EM algorithm. After calculation, we obtain the maximizer of the expectation of log-likelihood Λ(·) as follows:

(58) $$\Lambda \left(S_{t}=\left(A_{t},b_{t}\right)\right)=-A_{t}^{-1}b_{t},$$

where the sufficient statistics $S_{t}=\left(A_{t},b_{t}\right)$ include two additive-form statistics $A_{t}\left(x_{0:t}\right)\in R^{3\times 3}$ and $b_{t}\left(x_{0:t}\right)\in R^{3}$, given as follows:

(59) $$\begin{array}{c} A_{t}\left(x_{0:t}\right)=\sum_{\tau =1}^{t}{\left(\frac{\Delta t}{C_{m}}\right)}^{2}V_{\mathrm{ion}}\left(x_{\tau -1}\right)V_{\mathrm{ion}}^{⊤}\left(x_{\tau -1}\right), \end{array}$$

(60) $$\begin{array}{c} b_{t}\left(x_{0:t}\right)=\sum_{\tau =1}^{t}{\tilde{b}}_{\tau }\left(x_{\tau -1:\tau }\right)=\frac{\Delta t}{C_{m}}\left(v_{\tau }-v_{\tau -1}-\frac{\Delta t}{C_{m}}I_{\tau -1}\right)V_{\mathrm{ion}}\left(x_{\tau -1}\right). \end{array}$$

Here, $V_{\mathrm{ion}}\left(x_{t}\right)\in R^{3}$ represents the potentials of each ion channel, given as follows:

(61) $$V_{\mathrm{ion}}\left(x_{t}\right)=\left[\begin{array}{c} v_{t}-E_{L}\\ m_{\infty }\left(v_{t}\right)\left(v_{t}-E_{Ca}\right)\\ n_{t}\left(v_{t}-E_{K}\right) \end{array}\right].$$

#### 3.2.3. Formulation of the Augmented State-Space Model for Control

Here, we formulate the augmented SSM over the horizon. First, we derive the control transition of the augmented system model $p({\bar{u}}_{\tau +1}|\zeta_{\tau })$. In realistic scenarios, it is essential to guarantee that the net input current $I_{t}=u_{t}+I_{t}^{\mathrm{inj}}$ is always within a specific range. Therefore, we impose a norm constraint on the control input at every time *t* as follows:

(62) $$|u_{t}+I_{t}^{\mathrm{inj}}|\leq I_{\lim},$$

where $I_{t}^{\mathrm{inj}}$ is the current injected from other neurons and $I_{\lim}$ is the limit current. To this end, the control transition within the horizon is described as follows:

(63) $${\bar{u}}_{\tau +1}={\mathrm{clip}}_{I_{\lim}}\left({\bar{u}}_{\tau }+{\bar{I}}_{\tau +1}^{\mathrm{inj}}+z_{\tau +1}\right)-{\bar{I}}_{\tau +1}^{\mathrm{inj}},$$

where $z_{\tau +1}∼N\left(0,\sigma_{I}^{2}\right)$ is Gaussian noise. The proposed framework fixes the prediction of the external currents ${\bar{I}}_{\tau +1}^{\mathrm{inj}}$ at the value observed at time *t*, i.e., ${\bar{I}}_{\tau +1}^{\mathrm{inj}}=I_{t}^{\mathrm{inj}}$ for $\tau \in \left\{t,t+1,\ldots ,t+T_{H}-1\right\}$. Furthermore, the initialization for the control input particles is described as follows:

(64) $${\bar{u}}_{t}^{\left(i\right)}={\mathrm{clip}}_{I_{\lim}}\left(u_{t-1}^{\left(i\right)}+{\bar{I}}_{t}^{\mathrm{inj}}+z_{t}^{\left(i\right)}\right)-{\bar{I}}_{t}^{\mathrm{inj}},$$

where $z_{t}^{\left(i\right)}∼N\left(0,\sigma_{I0}^{2}\right)$ is Gaussian noise.

Next, we derive the augmented observation model for the augmented SSM over the horizon. We define the following augmented observation model to control the time series of membrane potentials toward a given reference trajectory $r_{t}\in R$:

(65) $$r_{t}∼N\left(r_{t}|v_{t},\sigma_{r}^{2}\right),$$

where $\sigma_{r}^{2}\in R$ is a variance that adjusts for acceptable error between the predictive state transition and the reference trajectory within the horizon.

#### 3.2.4. Settings

The true dynamics are assumed to follow the Morris–Lecar neuron model defined by Equation (53), and in simulation, they are solved using the Euler method. Here, the true parameters are those for the Homoclinic [37]. We apply our proposed method to the Morris–Lecar neuron model to control the membrane potentials $v_{t}$ toward the desired reference membrane potentials $r_{t}$. The reference trajectory $r_{t}$ is generated by the true model in advance. Note that we can only observe noisy membrane potentials; that is, we assume a partial observation situation.

The hyperparameters are set as follows. For simulation, the time step is Δt=0.1. The variances of the system noise and observation noise are $\Sigma_{x}=\mathrm{diag}\left(\sigma_{v}^{2},\sigma_{n}^{2}\right)=\mathrm{diag}\left(0.1^{2},0.001^{2}\right)$ and $\sigma_{y}^{2}=0.1^{2}$, respectively. For the PF, the number of particles is N=1000, and the threshold rate of resampling is α=0.8. The initial distribution for the initial particles $p_{0}\left(x\right)$ is $p_{0}\left(x\right)=N\left(v|\frac{2}{3}E_{L},10^{2}\right)U\left(n|0,1\right)$, where U(n∣0,1) is a uniform distribution within [0,1]. For parameter estimation, we use the threshold rate of backward resampling β=0.7 and the decay rate $\gamma_{t}=t^{-1}$. We update the parameters after $T_{\mathrm{burnIn}}=1000$ steps. To control the Morris–Lecar neuron model, we set the number of steps in the horizon as $T_{H}=10$. As a hyperparameter in the augmented SSM, the acceptable error variance and the control input limit are set to $\sigma_{r}^{2}=0.1^{2}$ and $I_{\lim}=150$, respectively. The variances of the augmented SSM’s control transitions are $\sigma_{I}^{2}=1^{2}$ and $\sigma_{I0}^{2}=10^{2}$.

#### 3.2.5. Results

**Figure 8:**
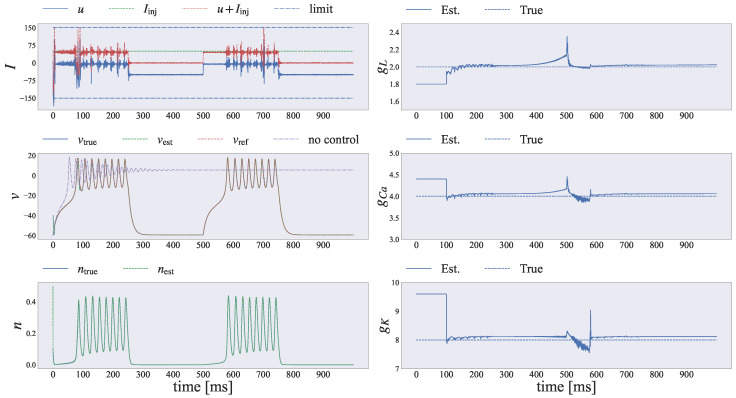
Results for simultaneously estimating and controlling the Morris–Lecar neuronal system. The dynamics of state variables $x_{t}={\left[v_{t},n_{t}\right]}^{⊤}$, control input *u*, and model parameters $\theta ={\left[g_{L},g_{Ca},g_{K}\right]}^{⊤}$ are shown. Top-left graph: Control current *u*, streaming currents $I_{\mathrm{inj}}$, and net input $u+I_{\mathrm{inj}}$ for the neuron, along with the control limit value. Middle-left graph: True membrane potential $v_{\mathrm{true}}$, estimated membrane potential $v_{\mathrm{est}}$, and reference trajectory $v_{\mathrm{ref}}$, along with the non-controlled trajectory. Bottom-left graph: True channel variable $n_{t}$ and estimated channel variable $n_{\mathrm{est}}$. Right three graphs: Estimated values of the parameters $g_{L},g_{Ca},g_{K}$, with true values indicated by dashed lines. We can simultaneously estimate and control the Morris–Lecar neuronal dynamics by applying our proposed method.

Figure 8 shows the simulation results after applying our proposed framework to the Morris–Lecar neuronal system. Here, we controlled the neuronal dynamics toward the reference trajectory, which represents the Homoclinic behavior during time intervals $0\leq t^{\prime }\leq 250$ and $500\leq t^{\prime }\leq 750$, and the resting state during intervals $250\leq t^{\prime }\leq 500$ and $750\leq t^{\prime }\leq 1000$. First, we found that our method could simultaneously estimate and control the Morris–Lecar neuron model. The middle-left graph shows the true membrane potential $v_{\mathrm{true}}$ (blue solid line), the estimated value $v_{\mathrm{est}}$ (green dashed line), and the target membrane potential $v_{\mathrm{ref}}$ (red dotted line). We found that the estimated value tracked the true value, as shown in the middle-left graph in Figure 8. In particular, the pale purple line represents the simulation results without adopting any control input. Without our proposed method, the Morris–Lecar neuron model converged to a specific value. However, by applying our proposed method, the Morris–Lecar neuron model was controlled toward the desired trajectory, allowing us to switch between neuronal firing and resting states. The bottom-left graph shows the true value $n_{\mathrm{true}}$ (blue solid line) and the estimated value $n_{\mathrm{est}}$ (green dashed line). We can see that the estimated value also closely tracked the true value.

Next, we verified whether our proposed method could estimate the parameters governing the dynamics of the neuron model. The right side of Figure 8 shows the parameters $\left(g_{L},g_{Ca},g_{K}\right)$ governing the Morris–Lecar neuronal model. As shown in the three graphs on the right, each estimated parameter value converged to the true value over time, although the estimated values spiked around 500 to 600.

Finally, we verified whether the proposed framework could estimate and control the neuronal dynamics under varying strengths of the control input $u_{t}$. The top-left graph shows the controllable input current *u* (blue solid line), the injected current $I_{\mathrm{inj}}$ (green dashed line), the actual net input $I=u+I_{\mathrm{inj}}$, and the limit value (light-blue dash-dotted line). The net input $u=u+I_{\mathrm{inj}}$ always satisfied the norm constraint $|u+I_{\mathrm{inj}}|\leq I_{\lim}$. Hence, we can simultaneously estimate and control the nonlinear neuronal dynamics while adhering to the constraint for the control signal.

## 4. Conclusions

In this paper, we have proposed a probabilistic framework for simultaneously estimating and controlling general dynamics entirely based on the particle filter. By introducing the particle filter not only as a state estimator and a prior estimator for the dynamics but also as a controller, we can directly handle the nonlinearity of the dynamics and the uncertainty of the latent state effectively and efficiently. With adaptive backward resampling, the proposed framework can suppress the degeneracy of the particles with respect to the sufficient statistics and realize accurate parameter estimation. Through experiments on two general models, including nonlinearity in distinct fields, we verified the effectiveness of our proposed framework. By applying our proposed framework to the Lorenz system in a simulation environment, we found that the proposed framework can not only estimate the latent variables from noisy observational data and the parameters governing chaotic behavior but also control the latent state toward the fixed point. Furthermore, by applying our proposed framework to the Morris–Lecar neuron model, we showed that even under noisy partial observation conditions, it can estimate the latent state and parameters and control the latent state toward the desired trajectory. Although our proposed method can simultaneously estimate the latent state and parameters and control nonlinear complex dynamics, our framework cannot handle semi-parametric or non-parametric models directly. In future work, we plan to extend our proposed framework to handle such semi-parametric or non-parametric models.

## Figures and Tables

**Table 1:** Input and output variables used in the Lorenz system.

	Variable	Description
Input	*u*	Control input
Output	x,y,z	Latent state
Output	σ,r,b	Parameters

**Table 2:** Input and output variables used in the Morris–Lecar neuron model.

	Variable	Description
Input	*I*	External input current
Output	v,n	Latent state
Output	$g_{L},g_{Ca},g_{K}$	Parameters

## Data Availability

The original contributions presented in the study are included in the article, further inquiries can be directed to the corresponding author (Toshiaki Omori).

## Abbreviations

The following abbreviations were used in this manuscript:

SSM	State-space model
PF	Particle filter
EM algorithm	Expectation-maximization algorithm
AdaSmooth	Adaptive smoothing
MPC	Model predictive control
PF-MPC	Particle filter-based model predictive control

## References

[1] Cheng S., Quilodrán-Casas C., Ouala S., Farchi A., Liu C., Tandeo P., Fablet R., Lucor D., Iooss B., Brajard J. (2023). Machine learning with data assimilation and uncertainty quantification for dynamical systems: A review. IEEE/CAA J. Autom. Sin..

[2] Inoue H., Hukushima K., Omori T. (2022). Estimating distributions of parameters in nonlinear state space models with replica exchange particle marginal metropolis-hastings method. Entorpy.

[3] Ito M., Kuwatani T., Oyanagi R., Omori T. (2021). Data-driven analysis of nonlinear heterogeneous reactions through sparse modeling and Bayesian statistical approaches. Entropy.

[4] Omori T., Kuwatani T., Okamoto A., Hukushima K. (2016). Bayesian inversion analysis of nonlinear dynamics in surface heterogeneous reactions. Phys. Rev. E.

[5] Ditlevsen S., Samson A. (2014). Estimation in the partially observed stochastic Morris–Lecar neuronal model with particle filter and stochastic approximation methods. Ann. Appl. Stat..

[6] Azza L.J., Crompton D., D’Eleuterio G.M.T., Skinner F., Lankarany M. (2023). Adaptive unscented Kalman filter for neuronal state and parameter estimation. J. Comput. Neurosci..

[7] Chan J.C., Strachan R.W. (2023). Bayesian state space models in macroeconometrics. J. Econ. Surv..

[8] Newman K., King R., Elvira V., de Valpine P., McCrea R., Morgan B.J.T. (2023). State-space models for ecological time-series data: Practical model-fitting. Methods Ecol. Evol..

[9] Ahwiadi M., Wang W. (2022). An enhanced particle filter technology for battery system state estimation and RUL prediction. Measurement.

[10] El-Dalahmeh M., Al-Greer M., El-Dalahmeh M., Bashir I. (2023). Physics-based model informed smooth particle filter for remaining useful life prediction of lithium-ion battery. Measurement.

[11] Kitagawa G. A Monte Carlo filtering and smoothing method for non-Gaussian nonlinear state space models. Proceedings of the 2nd U.S.-Japan Joint Seminar on Statistical Time Series.

[12] Doucet A., Freitas N., Gordon N. (2001). Sequenatial Monte Carlo Methods in Practice.

[13] Wills A.G., Schön T.B. (2023). Sequential Monte Carlo: A unified review. Annu. Rev. Control Robot. Auton. Syst..

[14] Kantas N., Doucet A., Singh S.S., Maciejowski J., Chopin N. (2015). On particle methods for parameter estimation in state-space models. Stat. Sci..

[15] Dempster A.P., Laird N.M., Rubin D.B. (1977). Maximum likelihood from incomplete data via the EM algorithm. J. R. Stat. Soc. Ser. B (Methodol.).

[16] Olsson J., Westerborn J. (2015). An efficient particle-based online EM algorithm for general state-space models. IFAC-Pap..

[17] Schwenzer M., Ay M., Bergs T., Abel D. (2021). Review on model predictive control: An engineering perspective. Int. J. Adv. Manuf. Technol..

[18] Stahl D., Hauth J. (2011). PF-MPC: Particle filter-model predictive control. Syst. Control Lett..

[19] Mastrototaro A., Olsson J., Alenlöv J. (2024). Fast and numerically stable particle-based online additive smoothing: The AdaSmooth algorithm. J. Am. Stat. Assoc..

[20] Särkkä S. (2013). Bayesian Filtering and Smoothing.

[21] Douc R., Cappe O. Comparison of resampling schemes for particle filtering. Proceedings of the 4th International Symposium on Image and Signal Processing and Analysis 2005.

[22] Hol J.D., Schon T.B., Gustafsson F. On resampling algorithms for particle filters. Proceedings of the 2006 IEEE Nonlinear Statistical Signal Processing Workshop 2006.

[23] Doucet A., Godsill S., Andrieu C. (2000). On sequential Monte Carlo sampling methods for Bayesian filtering. Stat. Comput..

[24] Neal R.M., Hinton G.E. (1998). A View of the EM Algorithm That Justifies Incremental, Sparse, and Other Variants.

[25] Cappé O., Moulines E. (2007). Online EM algorithm for latent data models. J. R. Stat. Soc. Ser. B Stat. Methodol..

[26] Olsson J., Westerborn J. (2017). Efficient particle-based online smoothing in general hidden Markov models: The PaRIS algorithm. Bernoulli.

[27] Omi T., Omori T. (2024). Simultaneously estimating and controlling nonlinear neuronal dynamics based on sequential Monte Carlo framework. Nonlinear Theory Appl..

[28] Lorenz E.N. (1963). Deterministic nonperiodic flow. J. Atmos. Sci..

[29] Pyragas K. (1992). Continuous control of chaos by self-controlling feedback. Phys. Lett. A.

[30] Pyragas V., Pyragas K. (2006). Delayed feedback control of the Lorenz system: An analytical treatment at a subcritical Hopf bifurcation. Phys. Rev. E.

[31] Shine J.M., Müller E.J., Munn B., Cabral J., Moran R.J., Breakspear M. (2021). Computational models link cellular mechanisms of neuromodulation to large-scale neural dynamics. Nat. Neurosci..

[32] Chialvo D.R. (2010). Emergent complex neural dynamics. Nat. Phys..

[33] Vogt N. (2019). Voltage imaging in vivo. Nat. Rev. Neurosci..

[34] Thomas K., Chenchen S. (2019). Optical voltage imaging in neurons: Moving from technology development to practical tool. Nat. Rev. Neurosci..

[35] Peterka D.S., Takahashi H., Yuste R. (2011). Imaging voltage in neurons. Neuron.

[36] Morris C., Lecar H. (1981). Voltage oscillations in the barnacle giant muscle fiber. Biophys. J..

[37] Ermentrout G.B., Terman D.H. (2010). Mathematical Foundations of Neuroscience.

